# Early pregnancy body mass index, gestational weight gain and perinatal outcome in an obstetric population in Lagos, Nigeria

**DOI:** 10.11604/pamj.2021.39.136.25926

**Published:** 2021-06-17

**Authors:** Olayinka Comfort Senbanjo, Fatimat Motunrayo Akinlusi, Tawaqualit Abimbola Ottun

**Affiliations:** 1 Department of Obstetrics and Gynaecology, Lagos State University Teaching Hospital, Ikeja Lagos State, Nigeria,; 2 Department of Obstetrics and Gynaecology, Lagos State University College of Medicine, Ikeja Lagos State, Nigeria

**Keywords:** Overweight, obesity, gestational weight gain, perinatal outcome, Nigeria

## Abstract

**Introduction:**

the burden of overweight and obesity is rapidly increasing worldwide with significant health and social consequences. We determined the prevalence of overweight and obesity, pattern of gestational weight gain (GWG) and the associations of these with perinatal outcome among pregnant women in Lagos, Nigeria.

**Methods:**

this was a retrospective review of case records of all deliveries in Lagos State University Teaching Hospital (LASUTH) over a period of two years. Case records of women with singleton pregnancies who registered for antenatal care at or below 20 weeks gestation were retrieved and reviewed to extract information on demography, anthropometrics, composites of pregnancy and perinatal outcomes. World Health Organization classification of BMI and the United States Institute of Medicine categorization of GWG were used to stratify subjects.

**Results:**

out of 4,512 deliveries, 365 (8.1%) met our criteria. The prevalence of overweight and obesity in early pregnancy was 34.6% and 25.6% respectively while 2.9% were underweight. Thirty-seven (11.1%) pregnant women gained more than the recommended weight while 77.8% of underweight pregnant women gained less than the recommended weight. Following multiple logistic regression analysis, obesity in early pregnancy was significantly related to hypertensive pregnancy disorder (AOR 2.2; 95% CI, 1.08-4.32, p = 0.030), gestational diabetes mellitus (AOR 14.4; 95% CI, 4.85-42.6, p = < 0.001), caesarean section (AOR 2.7; 95% CI, 1.51-4.87, p = 0.001) and infections (AOR 4.9; 95% CI, 1.93-12.62, p = 0.001) while excessive GWG was significantly associated with gestational diabetes mellitus (AOR 4.8; 95% CI, 1.63-14.12, p = 0.004).

**Conclusion:**

prevalence of early pregnancy overweight, obesity and excessive GWG were high among pregnant women in Nigeria and were associated with significant adverse consequences.

## Introduction

Recent reports indicate a worldwide increase in body mass index of the general population in the past few decades [1, 2]. The global mean prevalence of obesity in women has more than doubled in about four decades increasing from 6.4% in 1975 to 14.9% in 2014 and this has been projected to surpass 21% by 2025 if the trend should continue [2]. In low- and middle-income countries, under-nutrition is the usual nutritional problem while overweight and obesity are the commonest in developed countries. However, as a result of the change in lifestyle from consumption of traditional diet to the western diet and decreased physical activity, the prevalence of overweight and obesity are increasing in developing countries resulting in a ‘double burden’ of malnutrition [3, 4]. Globally, obesity is now a pandemic with serious medical, social and economic implications [1, 2, 4]. In addition, reports indicate that many women are entering pregnancy either overweight or obese and that the incidence of obesity and excessive weight gain during pregnancy is increasing with associated short and long term consequences on pregnancy and perinatal outcome [4]. In the United States of America [5], more than half of all pregnant women are obese while in Australia, 34% of pregnant women were overweight, obese or morbidly obese [6].

In a systemic review and meta-analysis, the prevalence of maternal obesity in Africa ranged from 6.5 to 50.7% with values varying between countries [4]. Nonetheless, studies have affirmed that the existing problem of maternal underweight remains prevalent in the world's poorest regions, especially in South Asia and sub-Sahara Africa [3]. Both insufficient and excessive Gestational Weight Gain (GWG) is strongly associated with maternal-foetal complications such as gestational diabetes mellitus (GDM), hypertensive pregnancy disorders (HPD), macrosomia, low birth weight, higher risk of caesarean deliveries and a higher incidence of anaesthetic and postoperative complications in these deliveries, low Apgar scores and neural tube defects are more frequent in infants of obese mothers than in infants of normal-weight mothers [7, 8]. In Nigeria, data from National Demographic Health Survey shows that the perinatal mortality rate is 41 deaths per 1000 pregnancies while the maternal mortality rate is 576 per 100,000 live birth which is the second highest in the world [9]. Most of these deaths are nutrition-related either directly or indirectly [3]. However, there is limited information on the effect of maternal obesity on pregnancy outcome among Nigerian women. Assessment of nutritional status in pregnancy is done at different times in the course of pregnancy using various tools. During the first or early trimester of pregnancy, the Body Mass Index (BMI) is widely recommended [10]. This study aimed to evaluate the BMI during the early stage of pregnancy, the pattern of weight gain during pregnancy and the influence of these on perinatal outcomes among women attending antenatal care service at the Lagos State University Teaching Hospital, Ikeja, Lagos, Nigeria.

## Methods

### Study design

The study was a retrospective review of case records to assess the nutritional status of pregnant women attending antenatal clinic (ANC) at the Lagos State University Teaching Hospital. The retrospective survey was for two years starting from 1^st^ January 2015 - 31^st^ December 2016.

### Study location

The study was conducted at the Maternal and Child Centers (MCC), Ifako-Ijaye, one of the two obstetrics units of LASUTH. The MCC is a 60-bed facility for maternal and neonatal care, Due to the policy of the state government, all the patients that present for antenatal care are booked resulting in an average of 400 deliveries per month. At booking, the patient's height and weight are usually recorded. The weight is taken with clients in their usual clothing to the nearest kg using a bathroom weighing scale while the height was measured using a height board to the nearest cm with the clients standing erect, without shoes, with eyes facing forward and feet together on the horizontal plane. The patients undergo routine antenatal visits and assessments every four weeks until the pregnancy is about 28 weeks, fortnightly until about 36 weeks gestation and thereafter weekly until delivery. Body mass index (BMI) was calculated using the formula: Weight (kg) / [Height (m)]^2^. For this study, the weight at booking was used for the calculation of early pregnancy body mass index. Gestational weight gain was defined as the difference between the maternal weight measured within one week prior to delivery and the maternal weight recorded at the first visit to the hospital.

### Ethical approval and consent to participate

The ethical approval for this study was obtained from the Research and Ethics Committee of the Lagos State University Teaching Hospital, Ikeja, Lagos State, Nigeria. The ethical approval reference number is LREC/06/10.1359.

### Study population

Pregnant women with singleton pregnancies who attended ANC at the MCC, Ifako-Ijaiye of Lagos State University Teaching Hospital were the participants. A booking antenatal visit before 20 weeks of pregnancy, deliveries in LASUTH and regular documentation of maternal height and weight were the inclusion criteria. Women with multiple gestations and those who booked beyond 20 weeks gestation were excluded from this study.

### Sampling technique

All the case notes of pregnant women attending ANC at the Maternal and Child Hospital, Ifako-Ijaye, one of the obstetrics units of LASUTH, were retrieved and reviewed for eligibility. A specifically designed study proforma was used to collect information on demographic characteristics of the pregnant women, gestational age at first antenatal care attendance, weight, height and body mass index of pregnant mother at booking, and weight of pregnant mother at the end of the second and third trimester, observed medical disorders, obstetric and perinatal complications. For the babies, information such as weight, Apgar score, need for Neonatal Unit admission was also recorded. Pregnant women who booked after 20 weeks and those with multiple gestations were excluded.

### Definition of terms

Nutritional status in pregnancy was determined at booking using BMI classification as defined according to WHO [11].

Weight gain in pregnancy was determined from serial weight measurements taken during antenatal visits. Weight gain during the first trimester was calculated from the difference between weight at 13 weeks gestation and weight measured at booking. Weight gain during the second trimester is the difference between weight at 26 weeks and weight at 13 weeks gestation while weight gain during the third trimester is the difference between weight just before delivery and weight at 26 weeks gestation. Overall gestational weight gain is the difference between weight measured at 36 weeks or just before delivery and weight measured at booking. The United States Institute of Medicine (IOM) revised gestational weight gain guidelines was used to determine the appropriateness of weight gain in pregnancy [12]. The recommended gestational weight gain for underweight, normal weight, overweight and obese pregnant women are 12.5 - 18, 11.5 - 16, 7 - 11.5 and less than 7kg respectively [12].

### Data analysis and presentation

The data obtained were entered into and analyzed using SPSS for Windows software version 22. Descriptive statistics were used to determine the associations between maternal obesity, excessive gestational weight and adverse pregnancy outcome using the Pearson Chi-squared (χ^2^) test or Fisher exact test for categorical variables as appropriate. Multiple regression analysis with adjustment for potential confounders was done for variables that had statistical significance on bivariate analysis, ‘p’ values of less than 0.05 was accepted as statistically significant.

## Results

Amongst 4512 deliveries, there were 365 women that met the inclusion criteria of booking for antenatal clinic at gestational age of less than or equal to 20 weeks during the two year study period. However, information was complete in only 344 women with regard to both weight and height records at booking and these women were studied and analyzed. The earliest gestational age at booking was 9 weeks while 21 (5.8%) pregnant women had their first ANC visit in the first trimester. Table 1 shows the socio-demographic characteristics and maternal body mass index of the study population. The mean age was 30.9 ± 4.2 years, with a minimum and a maximum age of 21 years and 43 years, respectively. About three-quarter (73.7%) of the women are in the age group of 25-34 years. About half (48.8%) of the women were nulliparous and 185 (61.5%) women had tertiary education. Body mass index during early pregnancy ranged from 13.6 to 49.4 kg/m^2^ with a mean value of 27.0 ± 5.4 kg/m^2^. The majority (60.2%) of the pregnant women were either overweight or obese.

**Table 1:** socio-demographic characteristics and body mass index classification of study population

Parameters	Frequency	Percentage (%)
**Age in years (n=339)**		
20-24	19	5.6
25-29	118	34.8
30-34	132	38.9
35-39	59	17.4
40-44	11	3.2
**Parity (n=340)**		
0	166	48.8
1	113	33.2
2	47	13.8
>3	14	4.1
**Tribe (n=336)**		
Yoruba	225	67.0
Ibo	44	13.1
Others	67	19.9
**Religion (n=336 )**		
Christian	292	86.9
Muslim	44	13.1
**Highest level of education (n=301)**		
No formal education	11	3.7
Primary	22	7.3
Secondary	83	27.6
Tertiary	185	61.5
**Body mass index classification (n=344)**		
<18.5 (Underweight)	9	2.6
18.5 - 24.9 (Normal)	128	37.2
25 - 29.9 (Overweight)	119	34.6
30 - 39.9 (Obesity)	80	23.3
40 - 49.9 (Morbidly obese)	8	2.3

Table 2 shows pattern of weight gain during pregnancy stratified by early pregnancy body mass index. Weight gain ranged from a minimum of 5.0 kg to maximum of 24.0 kg with a mean of 6.5 ± 4.2 kg. About half (54.1%) of the women gained less than the recommended weight for their early pregnancy body mass index. Relationships between early pregnancy BMI and pregnancy outcomes are shown in Table 3. Women with obesity during early pregnancy were significantly more likely to develop gestational hypertension (p = 0.010), gestational diabetes mellitus (p < 0.001), macrosomia (p =0.018), low birth weight (p =0.002), infections (p = 0.002) and to require caesarean section (p < 0.001) than women with normal early pregnancy BMI.

**Table 2:** pattern of weight gain during pregnancy stratified by early pregnancy body mass index

		Weight gain			
BMI	Frequency (%)	IOM guidelines (Kg)	Less than recommended	Recommended	More than recommended
**Underweight**	9 (2.6)	12.5-18	7 (77.8)	0 (0.0)	2 (22.2)
**Normal**	128 (37.2)	11.5-16	104 (83.9)	18 (14.5)	2 (1.6)
**Overweight**	119 (34.6)	7-11.5	69 (59.5)	36 (31.0)	11 (9.5)
**Obese**	88 (25.6)	<7	0 (0.0)	62 (73.8)	22 (26.2)
**Total**	344 (100.0)		180 (54.1)	116 (34.8)	37 (11.1)

BMI = body mass index; IOM = United States Institute of Medicine revised gestational weight gain guidelines (2009).

**Table 3:** relationship between early pregnancy body mass index and pregnancy outcome

Pregnancy outcomes		Early pregnancy BMI category				P value
	Frequency, N/344 (%)	Underweight	Normal	Overweight	Obese	
HPD (pre-eclampsia, eclampsia, PIH)	71 (20.6)	0 (0.0)	19 (26.8)	24 (33.8)	28 (39.4)	0.010
Cardiac disease	1(0.3)	0 (0.0)	0 (0.0)	1 (100.0)	0 (0.0)	0.594
Gestational diabetes mellitus	30 (8.7)	0 (0.0)	1 (3.3)	3 (10.0)	26 (86.7)	< 0.001
Caesarean section	133 (38.7)	3 (2.3)	33 (24.8)	44 (33.1)	53 (39.8)	< 0.001
Instrumental deliveries	2 (0.6)	0 (0.0)	1 (50.0)	0 (0.0)	1 (50,0)	0.728
Dystocia	24 (7.0)	1 (4.2)	7 (29.2)	7 (29.2)	9 (37.5)	0.505
Postpartum haemorrhage	11 (3.2)	0 (0.0)	3 (27.3)	3 (27.3)	5 (45.4)	0.470
Infections (UTI, Skin sepsis etc)	27 (7.8)	1 (3.7)	7 (25.9)	4 (14.8)	15 (55.6)	0.002
Maternal mortality	1 (0.3)	0 (0.0)	1 (100.0)	0 (0.0)	0 (0.0)	0.636
Stillbirth	7 (2.0)	0 (0.0)	2 (28.6)	3 (42.9)	2 (28.6)	0.918
Preterm delivery	24 (7.0)	0 (0.0)	10 (41.7)	8 (33.3)	6 (25.0)	0.846
Low birth weight	26 (7.6)	1 (3.8)	17 (65.4)	7 (26.9)	1 (3.8)	0.009
Macrosomia	30 (8.7)	0 (0.0)	7 (23.3)	10 (33.3)	13 (43.3)	0.036
Neonatal hypoglycemia	19 (5.5)	0 (0.0)	2 (10.5)	7 (36.8)	10 (52.6)	0.018
Neonatal admission	25 (7.3)	1 (4.0)	9 (36.0)	6 (24.0)	9 (36.0)	0.533

HPD - hypertensive pregnancy disorder; PIH - Pregnancy induced hypertension; UTI - urinary tract infection; Preterm delivery- babies delivered before 37 completed weeks of gestation; Low birth weight - babies with birth weight less than 2500 gram; Macrosomia - babies with birth weight equal to or more than 4000 gram; Neonatal hypoglycaemia- defined as plasma glucose level less than 40mg/dL.

Relationships between weight gain during pregnancy and pregnancy outcomes are shown in Table 4. Women with more than the recommended weight gain during pregnancy were significantly more likely to develop hypertension in pregnancy (p = 0.038), gestational diabetes mellitus (p < 0.001), macrosomic babies (p = 0.001) and have their babies develop neonatal hypoglycemia (p = 0.002) compared to women who gained recommended gestational weight. Women that gained less than recommended gestational weight had significantly higher proportion of low birth weight babies compared to women that gained recommended gestational weight (10.6% vs. 2.7%, p = 0.036). Results of multiple regression analysis (Table 5) after adjusting for confounders shows that early pregnancy obesity was associated with hypertensive pregnancy disorder (adjusted odds ratio 2.2; 95% CI, 1.08-4.32, p = 0.030), gestational diabetes mellitus (adjusted odds ratio 14.4; 95% CI, 4.85-42.6, p = < 0.001), caesarean section (adjusted odds ratio 2.7; 95% CI, 1.51-4.87, p = 0.001) and infections (adjusted odds ratio 4.9; 95% CI, 1.93-12.62, p = 0.001) while excessive gestational weight gain was only associated with gestational diabetes mellitus (adjusted odds ratio 4.8; 95% CI, 1.63-14.12, p = 0.004).

**Table 4:** relationship between gestational weight gain and pregnancy outcome

Pregnancy outcomes	Weight gain according to IOM guidelines			P value
	Less than recommended (n=180)	Recommended (n=116)	More than recommended (n=37)	
HPD (pre-eclampsia, eclampsia, PIH)	26 (14.4)	28 (24.1)	11 (29.7)	0.038
Cardiac disease	1 (0.6)	0 (0.0)	0 (0.0)	0.653
Gestational diabetes mellitus	3 (1.7)	17 (14.7)	9 (24.3)	< 0.001
Caesarian section	59 (32.8)	54 (46.6)	16 (43.2)	0.056
Instrumental deliveries	1 (0.6)	0 (0.0)	0 (0.0)	0.653
Dystocia	9 (5.0)	7 (6.0)	5 (13.5)	0.170
Postpartum haemorrhage	5 (2.8)	6 (5.2)	0 (0.0)	0.250
Infections (UTI, Skin sepsis etc)	11 (6.1)	11 (9.5)	4 (10.8)	0.449
Maternal mortality	1 (0.6)	0 (0.0)	0 (0,0)	0.651
Stillbirth	3 (1.7)	2 (1.7)	0 (0.0)	0.718
Preterm delivery	12 (6.7)	5 (4.3)	3 (8.1)	0.646
Low birth weight	19 (10.6)	4 (3.4)	1 (2.7)	0.036
Macrosomia	7 (3.9)	16 (13.8)	7 (18.9)	0.001
Neonatal hypoglycemia	3 (1.70	11 (9.5)	5 (13.5)	0.002
Neonatal admission	13 (7.2)	9 (7.8)	2 (5.4)	0.873

HPD - hypertensive pregnancy disorder; PIH - Pregnancy induced hypertension; UTI - urinary tract infection; Preterm delivery- babies delivered before 37 completed weeks of gestation; Low birth weight - babies with birth weight less than 2500 gram; Macrosomia - babies with birth weight equal to or more than 4000 gram; Neonatal hypoglycaemia-defined as plasma glucose level less than 40mg/dL

**Table 5:** multiple logistic regression analysis of pregnancy outcomes associated with early pregnancy obesity and excessive gestational weight gain

Parameters	Non-obese (n=256)	Obese (n=88)	AOR (95% CI)	P value
HPD (pre-eclampsia, eclampsia, PIH)	43 (16.8%)	28 (31.8%)	2.2 (1.08-4.32)	0.030
Gestational diabetes mellitus	4 (1.6%)	26 (29.5%)	14.4 (4.85-42.6)	0.000
Caesarean section	80 (31.3%)	53 (60.2%)	2.7 (1.51-4.87)	0.001
Infections (UTI, Skin sepsis etc)	12 (4.7%)	15 (17.0%)	4.9 (1.93-12.62)	0.001
Low birth weight	25 (9.8%)	1 (1.1%)	0.2 (0.04-1.12)	0.067
Macrosomia	17 (6.6%)	13 (14.8%)	1.03 (0.23-4.69)	0.967
Neonatal hypoglycaemia	9 (3.5%)	10 (11.4%)	1.1 (0.19-6.88)	0.896
**Parameters**	**Less than/recommended weight gain (n=296)**	**Excessive weight gain (n=37)**	**aOR (95% CI)**	**P value**
HPD (pre-eclampsia, eclampsia, PIH)	54 (18.2%)	11 (29.7)	1.3 (0.51-3.23)	0.594
Gestational diabetes mellitus	20 (6.8%)	9 (24.3)	4.8 (1.63-14.12)	0.004
Low birth weight	13 (4.4%)	1 (2.7)	0.6 (0.07-4.81)	0.622
Macrosomia	23 (7.8%)	7 (18.9)	0.8 (0.12-5.82)	0.849
Neonatal hypoglycaemia	14 (4.7%)	5 (13.5)	2.2 (0.29-17.0)	0.449

*Adjusted for maternal age, parity and gestational age at booking. 95% CI= 95% Confidence Interval; HPD - hypertensive pregnancy disorder; PIH - Pregnancy induced hypertension; UTI - urinary tract infection.

## Discussion

This study reviews the body mass index status during early pregnancy, maternal weight gain and the effect on pregnancy and perinatal outcomes among Nigerian women. In this study, the prevalence of overweight and obesity in early pregnancy was 34.6% and 25.6% respectively. This was higher than the prevalence of 10.7% in Enugu, Nigeria [13] and 9.6% in Benin City, Nigeria [14]. This difference might have resulted from the restriction of the sample population to pregnant women who booked for antenatal care before the first 13 weeks of gestation in these studies. However, it is instructive to note that the prevalence of overweight and obesity in this study was also higher than the prevalence of 27% overweight and 16% obesity reported for Australian women who were in the same range of gestational age with the Nigerian women in this study [15]. Australia together with other high-income countries currently suffers from the epidemic of obesity and non-commutable diseases. Our findings support that the prevalence of overweight and obesity among Nigerian women is high and that the problem of obesity is no longer that of developed countries alone but an emerging crisis in developing countries. This same trend has been quite visible in the Nigerian population in the last decade with estimates of the prevalence of overweight or obesity among adults in Nigeria ranging from 26% through 74.3% affecting approximately 40% of reproductive-aged women [16].This situation has been ascribed to high calorific intake, the nutritional transition to western type of diet and increased sedentary activities.

Appropriate weight gain during pregnancy is important for the well-being of both the mother and that of the baby. In this study, about half of the women gained less than the recommended gestational weight while one-tenth gained more than the recommended weight. Similar to the findings of Esimai *et al*. [17] and Bhavadharini *et al*. [18] in India, the majority of women with underweight, normal weight and overweight in early pregnancy ended up gaining less than recommended gestational weight. Among the obese pregnant women in this study, the majority gained less than the recommended 7 kilograms while about a quarter gained more than recommended gestational weight. The reason for the high prevalence of inappropriate gestational weight gain has been ascribed to the general belief by mothers that excessive weight gain during pregnancy may lead to difficult deliveries that may necessitate caesarean section [16].

In agreement with other studies [14, 15, 17, 18] this study has also shown that underweight, overweight and obesity during early pregnancy as well as excessive gestational weight gain were associated with adverse pregnancy and perinatal outcomes in Nigerian women. Specifically, overweight and obese Nigerian women have a significantly increased risk of gestational diabetes mellitus, caesarean section and infections and this is similar to reports from other centres both in developed and developing countries. Obese women may require interventions during pregnancy and delivery and these may account for a significantly high rate of caesarean section observed in this group of women compared to women with normal body mass index group. This is similar to the findings in Benin City, Nigeria where obese pregnant women had a higher rate of hospital admissions and caesarean section [14]. It is also similar to the findings in the province of Newfoundland and Labrador, Canada where the rate of caesarean section among women with extreme obesity was as high as 60% [19]. Caesarean section in obese women carries additional medical risks. Studies have shown potential difficulties with regional anaesthesia placement, increased risk of airway problems, deep venous thrombosis, and wound infection [20, 21]. In this study, obese women had significantly higher rates of infection. This may be because obesity predisposes to infection and obese women may experience wound complication after caesarean section. This is supported by a study that found a seven times risk of wound complications after caesarean section in women with obesity, some of whom required readmission and reoperation [22]. The amount of weight gained during pregnancy exerts an effect that is similar to body mass index status in early pregnancy. In this study, women that gained excessive weight also had higher rates of gestational diabetes mellitus and this is similar to findings in previous studies [18, 23].

This study has some limitations. The study was carried out in a tertiary hospital. This hospital is a major referral facility with a higher rate of adverse pregnancy outcomes compared with other hospital settings. Therefore, it may be difficult to generalize our findings to the broader community. The data in this study was collected retrospectively and may lead to a certain degree of inaccuracy. We recommend a longitudinal based research study and a larger sample frame to better understand the burden of obesity in pregnancy and the effect on adverse pregnancy outcome.

## Conclusion

In conclusion, this study shows that the prevalence of overweight and obesity in early pregnancy is high in the women studied. Under-nutrition which is a usual nutritional disorder in developing countries has a low prevalence. The study also shows a high rate of inappropriate weight gain during pregnancy among Nigeria women as only one-third of the women gained the recommended gestational weight. The high prevalence of overweight, obesity and excess weight gain during pregnancy among this group of Nigerian women were associated with gestational diabetes mellitus, caesarean section and infections. There is a need for an intervention that is aimed at educating all women in the reproductive age group about the importance of pre-conception body mass index and its effect on pregnancy and perinatal outcomes. There is also a need to create awareness about the importance of appropriate weight gain during pregnancy. The obstetricians and all the stakeholders must ensure that all pregnant women who come for antenatal care are classified into BMI categories according to WHO and counselled on appropriate weight gain. This will help in the early identification of women at risk of adverse pregnancy and perinatal outcomes and allow necessary interventional measures to be taken.

### What is known about this topic

- Under-nutrition is the usual nutritional problem throughout the life cycle in developing countries;
- The burden of overweight and obesity is rapidly increasing globally and the rate of increase is higher in developing than in developed countries.

### What this study adds

- The prevalence of obesity and excessive GWG is high among pregnant women in Lagos, Nigeria with a rate of about one out of every four and one out of every ten women respectively;
- Obesity and excessive GWG among pregnant women in Lagos, Nigeria are associated with significant adverse pregnancy and perinatal outcomes.
